# Effects of Hydrogen Peroxide on In Vitro Cultures of Tea (*Camellia sinensis* L.) Grown in the Dark and in the Light: Morphology, Content of Malondialdehyde, and Accumulation of Various Polyphenols

**DOI:** 10.3390/molecules27196674

**Published:** 2022-10-07

**Authors:** Evgenia A. Goncharuk, Maria Yu. Zubova, Tatiana L. Nechaeva, Varvara V. Kazantseva, Alexander A. Gulevich, Ekaterina N. Baranova, Petr V. Lapshin, Vera M. Katanskaya, Maria A. Aksenova, Natalia V. Zagoskina

**Affiliations:** 1 K.A. Timiryazev Institute of Plant Physiology, Russian Academy of Sciences, 127276 Moscow, Russia; 2 All-Russia Research Institute of Agricultural Biotechnology, Russian Academy of Sciences, 127550 Moscow, Russia; 3 N.V. Tsitsin Main Botanical Garden of Russian Academy of Sciences, 127276 Moscow, Russia

**Keywords:** hydrogen peroxide, polyphenols, in vitro, *Camellia sinensis* L.

## Abstract

Tea plants (*Camellia sinensis* L.) are phenol-accumulating crops that are widely used for public health. The healing effect of tea leaf products is due to the biosynthesis of such phenolic compounds (PCs) as flavans, which have P-vitamin capillary-strengthening activity. Due to their limited habitat and the value of their specialized metabolites of a phenolic nature, a promising approach is to establish in vitro cultures from them that retain the ability to form PCs, which is characteristic of ex vivo tea plants. The aim of this study was to investigate the effect of exogenic H_2_O_2_ (0.01 mM; 0.1 mM; 1 mM) on the growth, morphology, degree of stress response, and accumulation of various phenolic compounds in tea plant callus cultures of different ages (24 or 36 days) grown under different cultivation conditions (darkness or light). According to the results obtained, the H_2_O_2_ effect on tea callus cultures of different ages did not cause changes in their morphophysiological characteristics, both after 2 h of exposure (rapid response of callus culture, RRCC) and after 48 h (delayed response of callus culture, DRCC). The determination of the malondialdehyde (MDA) content, which serves as an indicator of changes in the level of lipid peroxidation (LPO) and the presence of stress responses in plant cells, indicated either its maintenance at the control level, a decrease, or an increase. All these effects depended on the growth conditions of the tea callus cultures (darkness or light), their age, the duration of exposure (rapid or delayed response), and the H_2_O_2_ concentration. Similar trends were noted for the total content of PCs as well as the amount of flavans, proanthocyanidins (soluble and insoluble forms), and lignin. The plant cell responses reflected changes in its adaptation programs, when specialized metabolites act as a target for the action of H_2_O_2_, thereby contributing to an increase in their resistance.

## 1. Introduction

The search for regulatory molecules that increase the resistance of plant cells to stress and the accumulation of biologically active substances (BAS) in them is of great interest to researchers [1,2,3]. These include hydrogen peroxide (H_2_O_2_), which acts as a regulator, terminator, or stimulator of cell growth [4,5]. Its participation in the processes of fruit ripening, leaf and flower formation, and the regulation of the functioning of photosystems, stomatal apparatuses, intracellular homeostasis, and other metabolic processes has been reported [6,7]. H_2_O_2_ is a messenger molecule that provides adaptive signal transduction and triggers a cascade of plant defense reactions under various stress conditions [8,9]. In addition, it can be involved in the inactivation of enzymes, damage to cell organelles, and the destruction of membranes, proteins, lipids, and nucleic acids, which can lead to the death of plant cells and tissues [10,11,12].

The effectiveness of H_2_O_2_ depends on its concentration and duration of exposure [13,14,15,16]. For example, low concentrations stimulated the formation of osmoprotectants and antioxidants, thus reducing the plant’s response to adverse environmental factors [17]. An excess amount of H_2_O_2_ increased the likelihood of the formation of hydroxyl radicals in the plant cells (the result of the Fenton reaction), which contributed to the induction of an “oxidative burst” in them and significant oxidative damage to cellular structures [12].

Phenolic compounds (PCs) or polyphenols are among the unique plant metabolites that, due to their chemical properties, are able to neutralize free radical processes, thereby protecting cells from the action of reactive oxygen species (ROS) [3,18,19,20]. They are synthesized in almost all cells and are extremely diverse in their structure, properties, and functional activity [21]. PCs can be monomeric (phenylpropanoids, flavonoids), oligomeric (proanthocyanidins), or polymeric (lignin). Some of them are widely represented in plants, while others are found only in certain families. In addition, PCs can be detected only in particular plant organs or at certain stages of ontogeny [22,23]. In most cases, the accumulation of PCs in aboveground organs is higher, and the composition is more diverse than in underground ones [24,25,26]. This is consistent with the idea that the biosynthesis of these metabolites is largely determined by the level of their intracellular differentiation, including the structural organization of plastids (chloroplasts) as one of the important sites of their biosynthesis in plant cells [27,28].

The growing interest in PCs is largely due to their antioxidant potential, which is manifested not only in plants, but also in the human organism [21,29]. They are considered biologically active plant metabolites exhibiting antiradical, antimutagenic, and anticarcinogenic activities [22,30]. The structural diversity and biological activity of PCs determines their uniqueness in the creation of many drugs, as well as the possibility of using them in the food, pharmaceutical, perfume, and cosmetic industries [19,31].

In vitro cultivated plant cells and tissues can be alternative sources of PC extraction, since biotechnological methods allow the product to be obtained regardless of changing external conditions while maintaining the natural habitats of valuable medicinal plants [32,33]. Despite the long period of application of such techniques, the production of secondary metabolites by this method faces a number of difficulties. Thus, rapidly growing dedifferentiated cell cultures with pharmacological activity, in most cases, does not retain the biosynthetic ability to form secondary metabolic products at a level characteristic of an intact plant [34,35]. In this case, it is advisable to optimize the cultivation conditions, which can be achieved by exposure to exogenous compounds, including H_2_O_2_, leading to the activation of signaling cascades and stimulating the biosynthesis of secondary metabolites, including PCs [36,37].

Tea plants, which are cultivated in many countries of the world, eaten, and used as a natural source of polyphenols, have been among the most economically important crops for centuries [38,39]. They are characterized by a high ability to accumulate a wide range of PCs, including gallic acid, simple and gallic forms of catechins, and proanthocyanidins [40,41,42]. The greatest accumulation of these substances is characteristic of young tea shoots. Moreover, special attention is paid to catechins due to their antioxidant, antibacterial, and antiallergic activities [43,44]. An analysis of the in vitro cell and tissue culture of the tea plant showed that in most cases, the accumulation of PCs and other valuable metabolites inherent in the tea plant decreased [45,46]. This was due to differences in the mechanisms of the biosynthesis of specialized metabolites in the in vitro plant system, with different levels of cell differentiation, and in intact plants. In particular, the dedifferentiated callus cells of the tea plant predominantly contained epicatechin and catechin, while catechins with a complex structure, such as epicatechin gallate and epigallocatechin gallate, were present only in the intact plant [45,47]. Cell proliferation and an increase in the level of their differentiation in the in vitro system did not contribute to an increase in the content of specialized metabolites or an improvement to their composition. Such a pattern has been noted in callus and suspension cultures of scarlet lychnis (*Lychnis chalcedonica* L.), white meadowsweet (*Spiraea betulifolia* L.), clinacanthus (*Clinacanthus nutans* Lindau), and other pharmacologically valuable plants that produce biologically active substances [48,49]. It is possible to stimulate an increase in this ability using various external influences, which was confirmed by the results obtained for many producing plants, including in vitro cell cultures of tea [45,50,51]. In this case, substances that act as triggers for the regulation of secondary metabolism, which can also be involved in the regulation of other processes in plant cells, are of most interest for study [52].

The aim of this study was to investigate the exogenous effect of H_2_O_2_ on the growth, morphology, level of stress response, and accumulation of various phenolic compounds in tea callus cultures grown under various cultivation modes (darkness or light).

## 2. Results

### 2.1. Morphophysiological Characteristics of Callus Cultures of the Tea Plant

**Figure 1:**
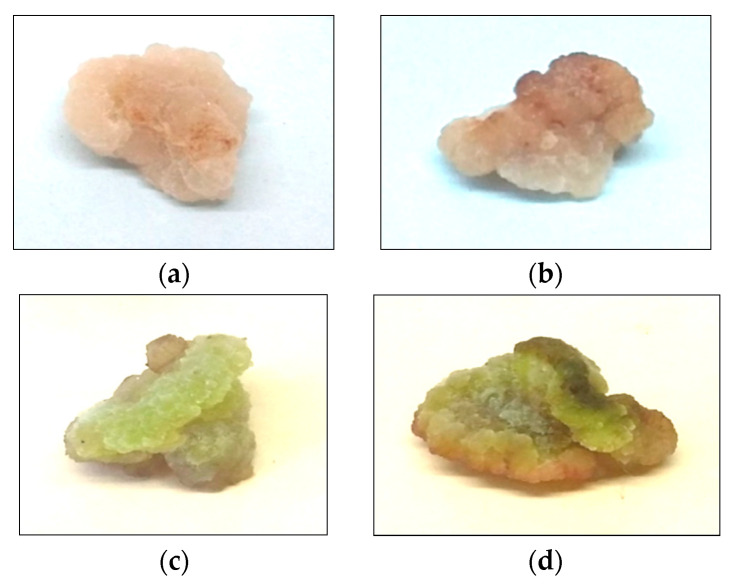
Established callus cultures initiated from stem explants of tea (*Camellia sinensis* L.) plants. Calli grown in the dark (**a**,**b**) or in the light (**c**,**d**) for 24 (**a**,**c**) or 36 (**b**,**d**) days.

The morphophysiological characteristics of the in vitro cultivated plant cells and tissues indicated their physiological state and response to exogenous factors [53]. Tea plant cultures of different ages grown under dark and light conditions differed in their morphophysiological parameters (Figure 1). When grown in the dark, they were dense and had a beige color, and they were lighter in a 24-day culture. Under light conditions, the calli were characterized by a slight greening, the intensity of which was higher in the 36-day-old cultures, which indicated the formation of chloroplasts in the cells. The presence of plastids that carried out full-fledged photosynthesis in some in vitro cultures of plant cells and tissues has been noted in a number of studies [54,55].

**Figure 2:**
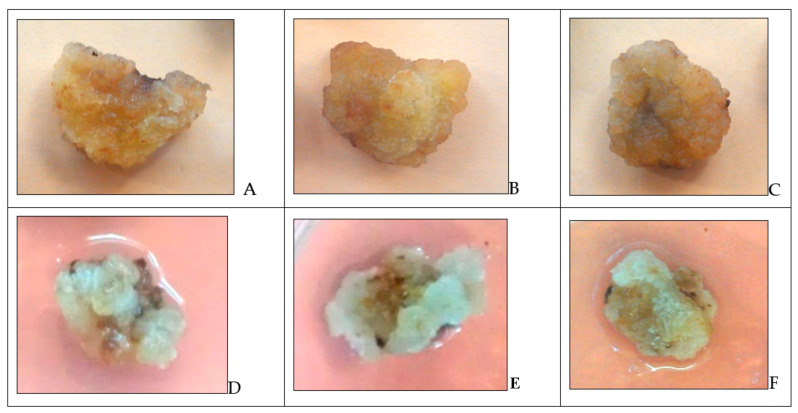
Tea callus culture at 24 days of age grown in the dark (**A**–**C**) or in the light (**D**–**F**) and kept in water (**A**,**D**) or 1 mM H_2_O_2_. (**A**,**D**) control; (**B**,**E**) short-term exposure to H_2_O_2_ (2 h); (**C**,**F**) aftereffect of H_2_O_2_ (after 48 h).

Under the RRCC on the influence of various concentrations of H_2_O_2_, no changes in the morphological characteristics of tea callus cultures of different ages were observed (Figure 2). Another trend was noted with the DRCC, where in all variants, the calli acquired a darker color; the efficiency of this process was concentration-dependent and was more pronounced at high doses of H_2_O_2_.

The determination of the biomass increment in these calli did not reveal any changes during the RRCC and DRCC, which may have been due to the low growth activity of the tea culture [56].

An important indicator of the state of plant cells and tissues is their water content [57]. In tea calli of different ages grown in the dark, this parameter was 92–94%, and when grown in the light, it was 90–92%. After exposure to H_2_O_2_, their water content decreased relative to the control by 5–10%, which was more pronounced in 36-day-old cultures with the DRCC. This indicates that the exogenous action of this molecule on tea callus cultures of different ages grown in the dark and in the light did not cause changes in their morphophysiological characteristics.

### 2.2. Malondialdehyde Content in Callus Cultures of the Tea Plant

The processes of lipid peroxidation (LPO) are characteristic of all plant cells, and their intensity depends on many factors, including age, growing conditions, exogenous influences, and the level of cellular differentiation [12,58]. An increase in the MDA content, as one of the main indicators of the LPO level, is due to a change in the balance between the formation and scavenging of ROS and is considered one of the criteria for the presence of a stress response in plant cells [36,59].

**Figure 3:**
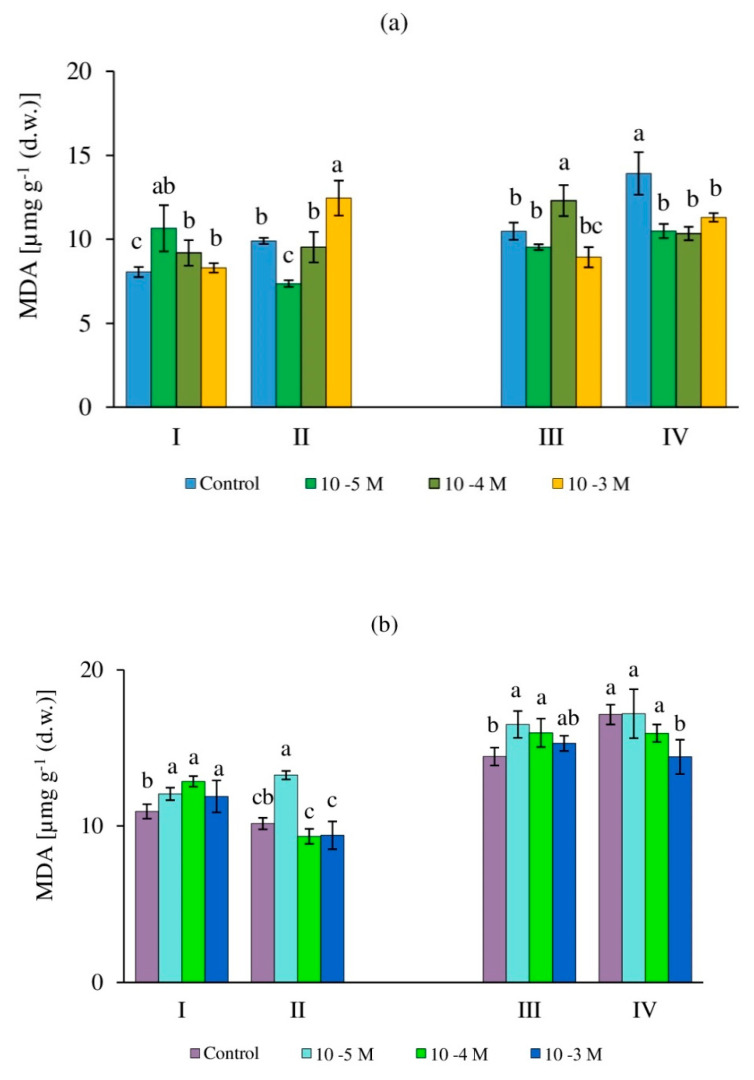
Content of malondialdehyde (MDA) in callus cultures of tea plants 24 days old (I, II) and 36 days old (III, IV) grown in the dark (**a**) or in the light (**b**) and exposed to various concentrations of H_2_O_2_—0 (control), 0.01 mM, 0.1 mM, and 1 mM. Variants: I, III—rapid response of callus cells (2 h exposure to H_2_O_2_); II, IV—delayed response of callus cells (48 h after exposure to H_2_O_2_). Different letters indicate significant differences between exposure conditions (*p* ≤ 0.05).

The determination of the MDA content in tea calli grown in the dark showed that the effect of various H_2_O_2_ concentrations on 24-day-old cultures did not cause significant changes in most cases (Figure 3a). An exception was observed with the RRCC on 0.01 mM H_2_O_2_ and the DRCC on 1 mM H_2_O_2_, where the MDA was noted to increase by 20% and 30% relative to the control, respectively. In a 36-day-old callus culture, the amount of MDA in the control variant was insignificant (by 8%), but statistically significantly higher than that of the earlier growth period (day 24). At the same time, there were no changes in this indicator with the RRCC on 0.01 mM and 1 mM H_2_O_2_, and the response on 0.1 mM H_2_O_2_ increased (by 20% relative to the control). A different trend was observed with the DRCC, when the highest content of MDA was noted in the callus of the control variant, and in all experimental variants it was equal to one another (DRCC), significantly lower respectively to the control variant (DRCC) (on average by 23%) and close to the values with the RRCC, which indicated no changes to the antioxidant system functioning in tea cultures during the completion of their linear growth.

The tea callus culture grown under light conditions was characterized by a higher content of MDA compared to that of the culture grown in the dark (Figure 3b). At the culture ages of 24 and 36 days, these differences were 30% and 25%, respectively. The amount of MDA increased statistically significantly in a 24-day callus culture with the RRCC in all experimental variants and equally (by 9%). With the DRCC, this indicator was lower in all variants (by 8% on average), except for the response to 0.01 mM H_2_O_2_, where it was the highest and equal to that with the RRCC of a 24-day-old callus culture. At a later stage of growth of tea calli (36 days), the content of MDA in all variants was almost 30% higher than the values of the earlier period of their growth (24 days), as was also noted for calli grown in the dark. With the RRCC of 36-day callus cultures grown in the light, the MDA content statistically significantly increased (by 5–8% relative to the control) under the influence of various concentrations of H_2_O_2_. With the DRCC, this indicator was in most cases equal both in the control and experimental variants, with the exception of exposure to 1 mM H_2_O_2_, where the MDA content was lower (by 15%). A decrease in the MDA content may be a consequence of the antioxidant system functioning, which prevents the processes of oxidative damage to cellular structures.

### 2.3. The Content of Phenolic Compounds in Callus Cultures of the Tea Plant

PCs are unique and widespread specialized plant metabolites with antioxidant activity [19]. The accumulation of these compounds is species- and organ-specific and depends on growth conditions and the impact of exogenous factors [18,60].

**Figure 4:**
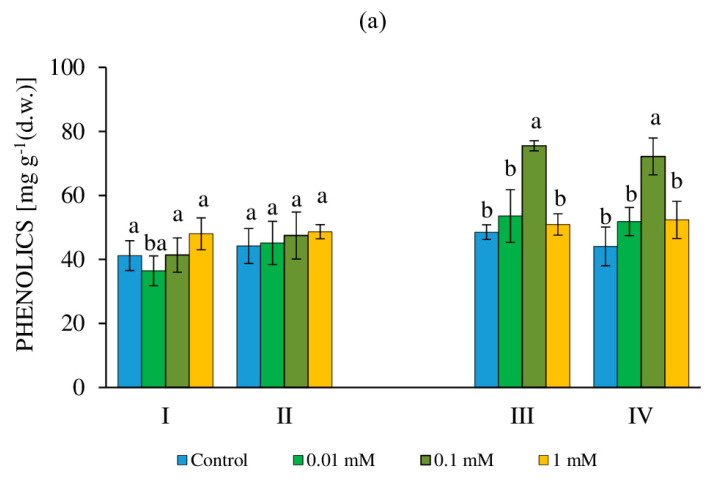

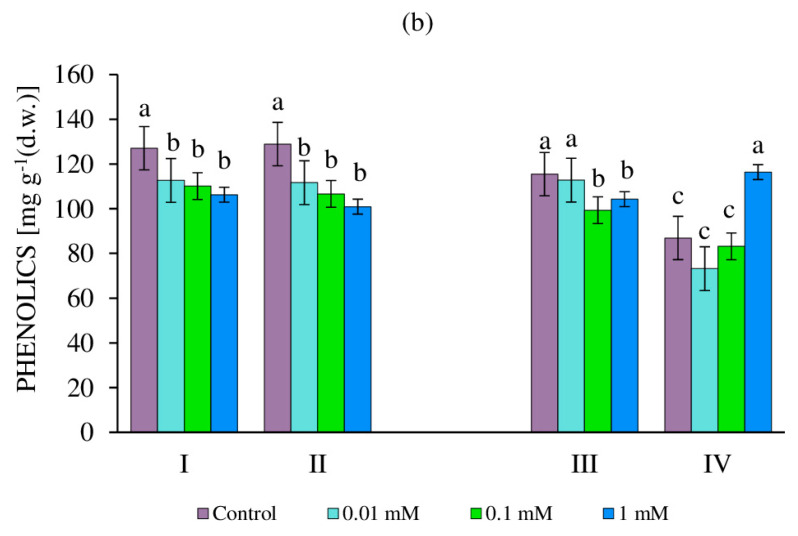
The content of the total phenolic compounds in callus cultures of tea plants at 24 days (I, II) and 36 days (III, IV) of age, grown in the dark (**a**) or in the light (**b**) and exposed to various concentrations of H_2_O_2_—0 (control), 0.01 mM, 0.1 mM, and 1 mM. Variants: I, III—rapid response of callus cells (2 h exposure to H_2_O_2_); II, IV—delayed response of callus cells (48 h after exposure to H_2_O_2_). Different letters indicate significant differences between exposure conditions (*p* ≤ 0.05).

Tea callus cultures grown in the dark or in the light differed in their total PC content and response to H_2_O_2_ exposure (Figure 4). A higher (3–4 times) accumulation of these metabolites was noted during their cultivation under light conditions, which may be due to the light regulation of the activity of some enzymes of phenolic metabolism [18].

In tea callus cultures grown in the dark, the content of the total PCs on day 24 was lower compared to that on day 36, and these differences reached 10% (Figure 4). They also differed in their response to the exposure to different concentrations of H_2_O_2_. Thus, in 24-day-old calli, there were no statistically significant changes in the content of PCs with the RRCC and DRCC (Figure 4a). In 36-day-old callus cultures, the PC content increased with the RRCC and DRCC, although it was statistically significant only with a 0.1 mM H_2_O_2_ exposure (by 55% and 63% relative to the control, respectively).

The callus culture of tea grown in the light showed a different trend (Figure 4b). In 24-day-old calli, the total PC content decreased both with the RRCC (by 11–19%) and with the DRCC (by 16–21%), and this effect was more pronounced at high H_2_O_2_ concentrations. In 36-day-old calli with the RRCC, a decrease in the PC level was also noted, but this effect was manifested only with 0.1 and 1 mM H_2_O_2_ (by 15% and 10% relative to the control, respectively). In the callus tea cultures of the control and most experimental variants, the amount of PCs with the DRCC was lower relative to that with the RRCC (by 30–40%), and there was also no reaction to the effect of H_2_O_2_. An exception was the variant with 1 mM H_2_O_2_, in which case this indicator exceeded the values of all other variants (by 33%).

All this suggests differences for the RRCC and DRCC on the influence of H_2_O_2_ in tea callus cultures of different ages grown in the dark and in the light.

### 2.4. The Content of Flavans in Callus Cultures of the Tea Plant

**Figure 5:**
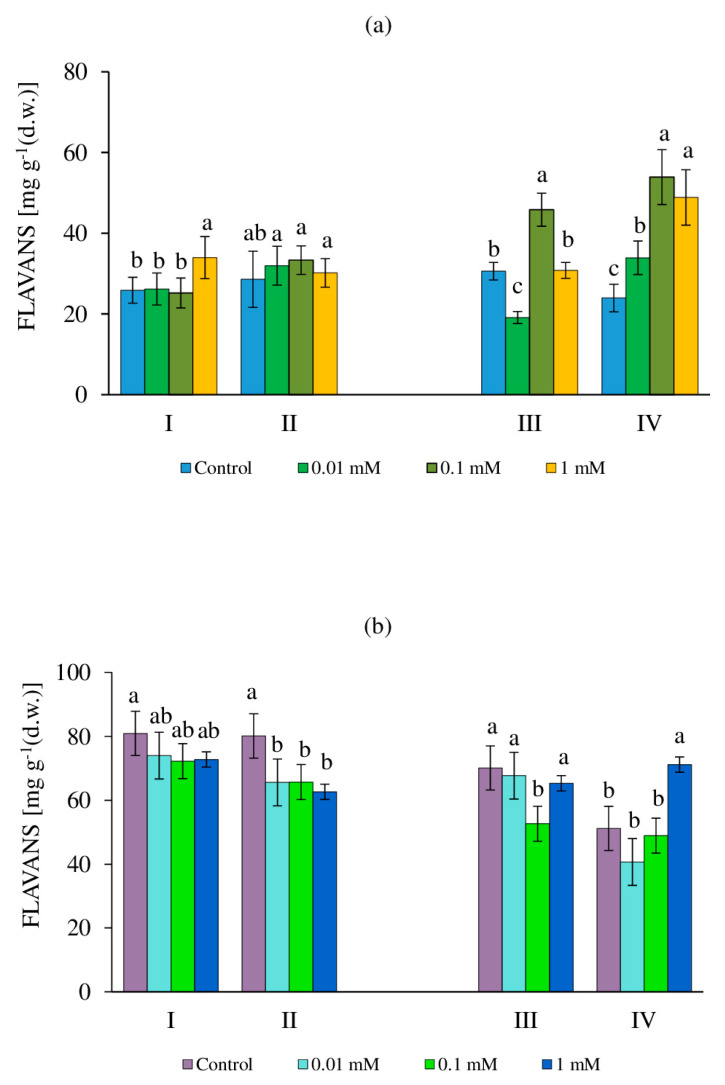
The content of flavans (FLs) in callus cultures of tea plants at 24 days (I, II) and 36 days (III, IV) of age, grown in the dark (**a**) or in the light (**b**) and exposed to various concentrations of H_2_O_2_—0 (control), 0.01 mM, 0.1 mM, and 1 mM. Variants: I, III—rapid response of callus cells (2 h exposure to H_2_O_2_); II, IV—delayed response of callus cells (48 h after exposure to H_2_O_2_). Different letters indicate significant differences between exposure conditions (*p* ≤ 0.05).

Flavonoids are one of the most abundant specialized plant phenolic metabolites that have high antioxidant activity closely related to their redox properties [61,62]. One type of flavonoids in tea plants are flavans (FLs)—unique substances with P-vitamin capillary-strengthening activity [56,62]. Their content in tea callus cultures grown in the light was 1.5–2.9 times higher than that in cultures grown in the dark (Figure 5). These differences depended both on their age and the application of various concentrations of H_2_O_2_.

In 24-day-old tea callus cultures grown in the dark, the FL content in most cases did not change relative to the control during the RRCC with the influence of various H_2_O_2_ concentrations (Figure 5a). An increase (by 30%) with 1 mM H_2_O_2_ was an exception, which indicated changes in the accumulation of these metabolites at high concentrations. The content of FLs in both the control and experimental variants with the DRCC was equal, which indicated the absence of changes in their formation. A different trend was observed in 36-day callus cultures. With the RRCC, the content of FLs in the cultures decreased by 40% relative to the control under the action of 0.01 mM H_2_O_2_, increased with 0.1 mM H_2_O_2_ (by 30%), and did not change with 1 mM H_2_O_2_. With the DRCC, the amount of these metabolites in callus cultures increased relative to the control: with 0.01 mM H_2_O_2_, by 30%, and with higher concentrations (0.1 mM and 1 mM H_2_O_2_), by almost two times. This indicated a stimulating post-effect of H_2_O_2_ in relation to the accumulation of these representatives of phenolic metabolism in the tea callus culture grown in the dark for 36 days (the end of the linear growth phase).

Another trend is typical for tea cultures grown in the light (Figure 5b). In 24-day-old calli with the RRCC, the effect of H_2_O_2_ showed an almost equal decrease in the amount of FLs (by 7–8% relative to the control), although these differences were statistically significantly small. A similar trend was noted with the DRCC, but these changes were more pronounced (by 18% relative to the control). In 36-day-old calli with the RRCC, the FL content remained at the control level under the influence of 0.01 and 0.1 mM H_2_O_2_, in contrast to 0.1 mM H_2_O_2_, where the level was 25% lower. The amount of these metabolites with the DRCC in most of the studied variants was equal to or 30–50% lower than in the earlier study period (RRCC). An exception was the variant with 1 mM H_2_O_2_, where the FL amount was maximized.

### 2.5. The Content of Free and Bound Proanthocyanidins in Callus Cultures of the Tea Plant

Proanthocyanidins (PAs) are derivatives of flavan-3-ols and flavan-3,4-diols of various degrees of polymerization [42]. Their synthesis is a characteristic of intact tea plants and established callus cultures [56,63].

**Figure 6:**
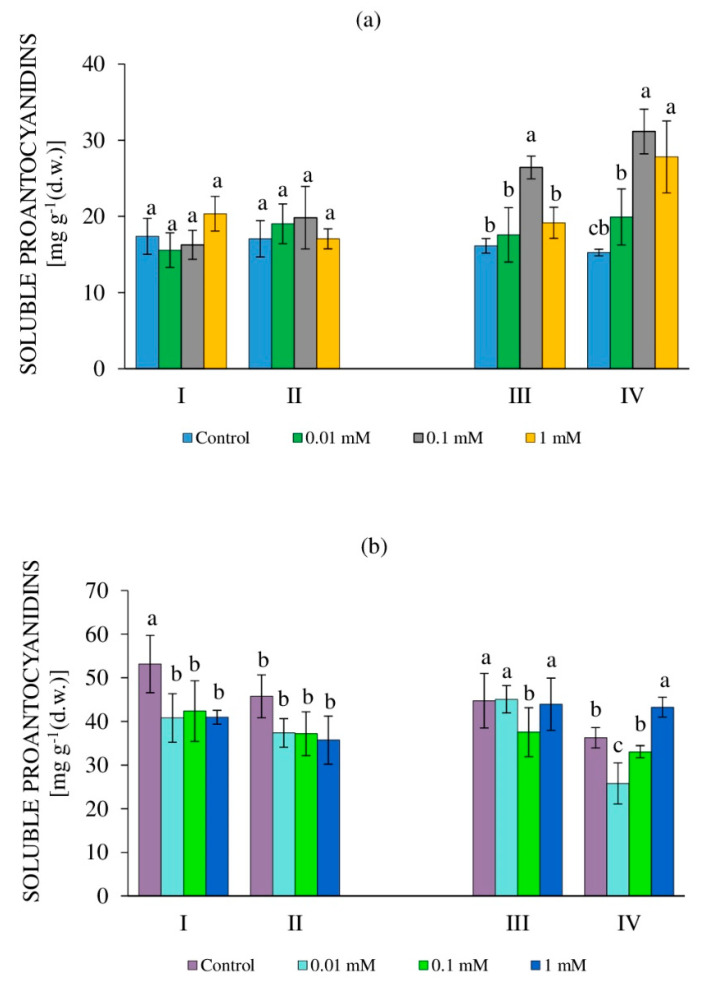
The content of soluble proanthocyanidins in callus cultures of tea plants at 24 days (I, II) and 36 days (III, IV) of age, grown in the dark (**a**) or in the light (**b**) and exposed to various concentrations of H_2_O_2_—0 (control), 0.01 mM, 0.1 mM, and 1 mM. Variants: I, III—rapid response of callus cells (2 h exposure to H_2_O_2_); II, IV—delayed response of callus cells (48 h after exposure to H_2_O_2_). Different letters indicate significant differences between exposure conditions (*p* ≤ 0.05).

The determination of soluble PA (SPA) content in 24-day-old tea callus cultures grown in the dark and exposed to H_2_O_2_ did not reveal significant changes in this indicator with the RRCC and DRCC (Figure 6a). In most cases, it was equal to that of the control, although a slight increase can be noted with the RRCC upon exposure to a high concentration (1 mM) of H_2_O_2_. In 36-day-old calli grown under these conditions, with the RRCC, the content of SPAs remained at the control level with 0.01 and 1 mM H_2_O_2_ and increased with 0.1 mM H_2_O_2_ (by 40%). The SPA accumulation in the calli of the control variant and with 0.01 mM H_2_O_2_ was the smallest with the DRCC and equal to that with the RRCC. For other variants (0.1 mM and 1 mM H_2_O_2_), the SPA content was statistically equal and high, exceeding the control values by almost twice.

In tea callus cultures grown in the light, the content of SPAs was several times higher than that in cultures grown in the dark (Figure 6). At the same time, in 24-day-old calli exposed to various concentrations of H_2_O_2_, the SPA content decreased to an equal extent relative to the control for both the RRCC and DRCC (by an average of 25%) (Figure 6b). The response of 36-day-old cultures to the application of H_2_O_2_ was similar. The amount of SPAs in most of the studied variants with the RRCC was equal, except for 0.1 mM H_2_O_2_, where it decreased by 16% relative to the control. The accumulation of these metabolites with the DRCC in the calli of the control variant and under exposure of 0.1 mM H_2_O_2_ was equal, with 0.01 mM H_2_O_2_ it was lower (by 25%), and with 1 mM H_2_O_2_ it was higher (by 19%) and almost equal to that of the RRCC. Consequently, the effect of exposure to a high concentration of H_2_O_2_ (1 mM) on the accumulation of SPAs in the tea callus culture grown in the light for 36 days was preserved during its further cultivation, which is not typical for lower concentrations, when the level of these metabolites further decreased. Perhaps these changes are the result of the “redistribution” of the metabolites to other cellular compartments, for example, to the cell wall [56].

**Figure 7:**
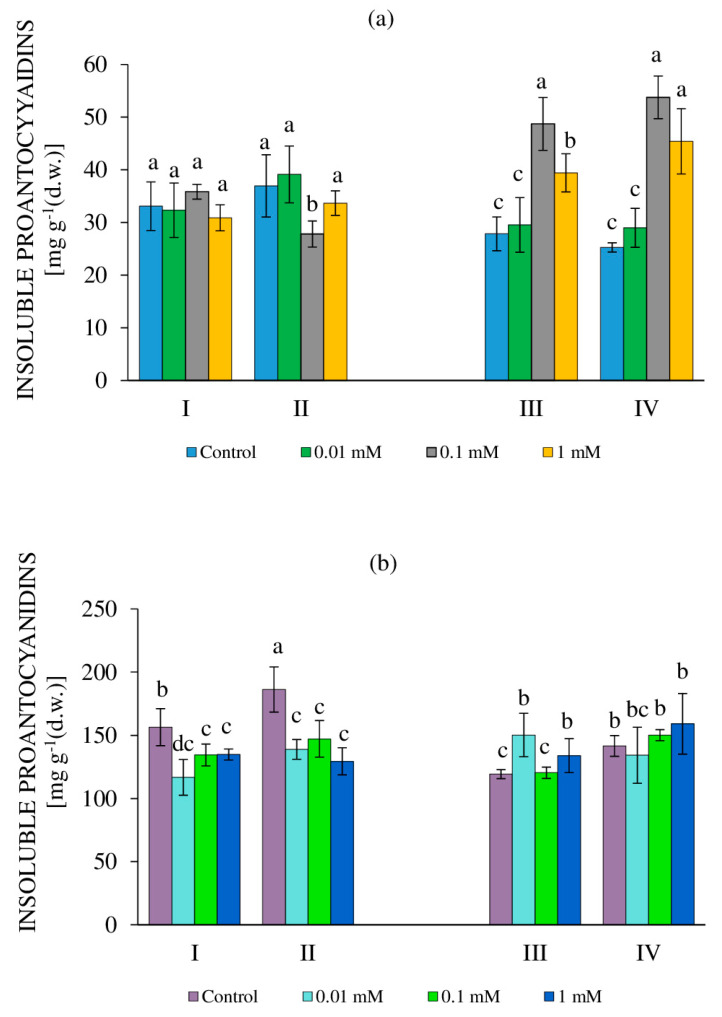
The content of insoluble (bound) proanthocyanidins in callus cultures of tea plants at 24 days (I, II) and 36 days (III, IV) of age, grown in the dark (**a**) or in the light (**b**) and exposed to various concentrations of H_2_O_2_—0 (control), 0.01 mM, 0.1 mM, and 1 mM. Variants: I, III—rapid response of callus cells (2 h exposure to H_2_O_2_); II, IV—delayed response of cells (36 h after exposure to H_2_O_2_). Different letters indicate significant differences between exposure conditions (*p* ≤ 0.05).

It is known that PAs accumulate in plant cells, both in a free state and in a form bound to cell organelles [64,65]. As we noted earlier, in the heterotrophic tea culture, the content of bound (insoluble) forms of PAs (IPAs) was 2–4 times higher than that of SPAs [56]. This trend is also characteristic of the tea cultures used in our study, grown in the dark and in the light (Figure 7).

Tea callus cultures of different ages grown in the dark differed in their response to the action of H_2_O_2_ (Figure 7a). In 24-day-old calli with the RRCC, the content of IPAs in all variants was equal to that of the control. A similar trend was observed to a certain extent with the DRCC, except for a lower amount with 0.1 mM H_2_O_2_. All this suggests the absence of changes in the accumulation of IPAs under the influence of various concentrations of H_2_O_2_ on 24-day-old tea callus cultures. In 36-day-old calli, the amount of these metabolites with the RRCC in the control variant and with a 0.01 mM H_2_O_2_ exposure was equal, and with 0.1 and 1 mM H_2_O_2_ it exceeded the control values by 50% and 40%, respectively. This effect was also preserved with the DRCC and was even more pronounced. Therefore, the exposure of 36-day-old tea callus cultures grown in the dark to high concentrations of H_2_O_2_ contributed to the accumulation of IPAs in the calli.

In the tea callus cultures grown in the light, the content of IPAs was 4–5 times higher than that in the cultures grown in the dark (Figure 7b). In 24-day-old calli with the RRCC and DRCC, the IPA content under the influence of all concentrations of H_2_O_2_ was equal to and lower than the control values by 17% and 28%, respectively to RRCC and DRCC. A different trend was noted in 36-day-old callus cultures, in which the IPA content remained unchanged in all variants, which indicated the stable accumulation of IPAs in calli and the absence of a response to the application of H_2_O_2_.

### 2.6. The Content of Lignin in Tea Callus Cultures Grown in the Dark or in the Light

Lignin is a polymer of a phenolic nature, the formation of which is characteristic of almost all plant cells [43]. In plant cell cultures, its biosynthesis does not occur in all cases, although it is carried out in tea calli [24].

**Figure 8:**
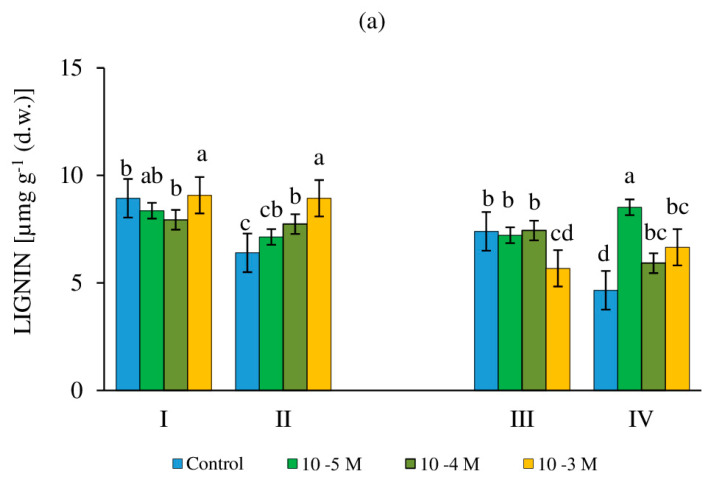

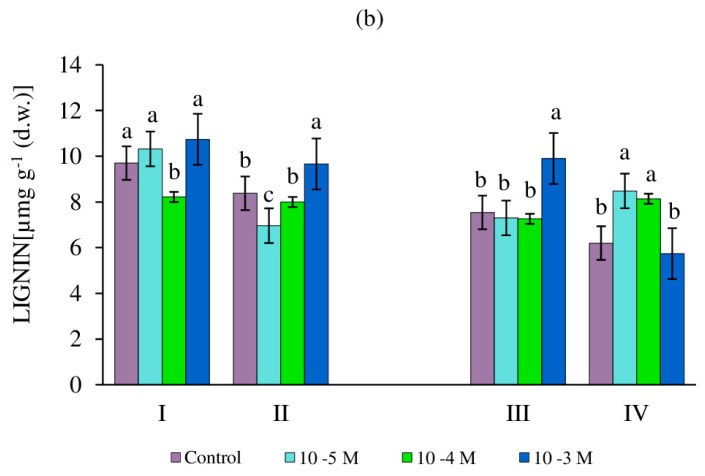
The content of lignin in callus cultures of tea plants at 24 days (I, II) and 36 days (III, IV) of age, grown in darkness (**a**) or light (**b**) and exposed to various concentrations of H_2_O_2_—0 (control), 0.01 mM, 0.1 mM, and 1 mM. Variants: I, III—rapid response of callus cells (2 h exposure to H_2_O_2_); II, IV—delayed response of callus cells (48 h after exposure to H_2_O_2_). Different letters indicate significant differences between exposure conditions (*p* ≤ 0.05).

Callus cultures of tea grown in the dark and in the light were similar in lignin content (Figure 8). The amount of lignin in most cases was higher on the 24th day of growth, and then slightly decreased by the 36th day.

In tea calli of different ages grown in the dark, the lignin content in most cases did not change relative to the control with the RRCC upon exposure to various concentrations of H_2_O_2_ (Figure 8a). A decrease in the lignin content was noted in two cases: in a 24-day culture with 0.1 mM H_2_O_2_, and in a 36-day culture with 1 mM H_2_O_2_ (by 10% and 23%, respectively). The lignin content of 24-day-old calli with the DRCC was lower than that with the RRCC: by 27% for the control, within 10% for 0.01 and 0.1 mM H_2_O_2_, and it did not change for 1 mM H_2_O_2_ (Figure 8a). In 36-day-old calli, the lignin content was low in the control variant with the DRCC, and this value was exceeded under the action of H_2_O_2_, especially with 0.01 mM H_2_O_2_ (by 82%).

In a 24-day-old tea callus culture grown in the light, no statistically significant changes in the lignin content were observed with the RRCC on the various H_2_O_2_ concentrations (Figure 8b). Its accumulation decreased with the DRCC relative to RRCC (in control and with 0.01 mM H_2_O_2_ by 15% and 30%, respectively) or remained at the same level. The highest amount of lignin among these variants was noted after exposure to 1 mM H_2_O_2_. In 36-day-old cultures with the RRCC, in most cases it was equal to that of the control. An exception was the variant with 1 mM H_2_O_2_, where the lignin content was 30% higher. Lignin amount exceeded the control values with the DRCC: with 0.01 mM and 0.1 mM H_2_O_2_ by 31% and 36%, respectively, and with 1 mM H_2_O_2_ it corresponded to control value.

All this indicates differences in the formation of lignin in tea callus cultures of different ages grown in the dark and in the light, as well as their response to the exogenous application of H_2_O_2_.

## 3. Discussion

Hydrogen peroxide (H_2_O_2_) is an integral component of the metabolism of plant cells and tissues. The main source of its formation is photorespiration, the functioning of electron transport chains, and redox reactions in the apoplast [9,17]. A participant in this process, molecular oxygen, is subjected to enzymatic reduction to H_2_O_2_ by a stepwise reaction with the participation of an intermediate superoxide anion (O^2–^). It should be noted that this molecule is a chemical oxidant that has proven to be a key participant in the response of plant cells to stress and their programmed death [10,11,12,59]. The role of H_2_O_2_ as a signaling molecule that “triggers” a cascade of plant defense reactions is also considered [8,9]. All this testifies to its diverse regulatory role in the vital activity of plants. Recently, more and more studies have investigated the use of H_2_O_2_ as a growth regulator and adaptogen in the cultivation of various crops, both in vivo and in vitro [5,66]. In the latter case, plant cell cultures are of particular interest as producers of pharmacologically valuable secondary metabolites, including phenolic ones [1].

It is known that the effectiveness of H_2_O_2_ action is due to many factors, including its concentration, duration of exposure, the physiological state of the cells, the method of processing, and the aftereffect, as well as the endogenous content of certain classes of metabolites, which includes PCs [15,16,19]. Due to their chemical properties and interaction with this molecule, PCs prevent free radical processes in cells and prevent the development of the so-called oxidative explosion, which even leads to cell death [3,20]. The structure of PCs is extremely diverse, which affects their properties and functional activity [21].

According to the literature data, H_2_O_2_ concentrations ranging from 0.01 mM to 1 mM are the most frequently applied by researchers for in vitro cultures [5,8,13]. This is related to the characteristics of this compound, which leads to oxidative stress and is known to be cytotoxic when it is used in concentrations higher than those mentioned above. However, H_2_O_2_ may have a cytoprotective effect within the specified concentration limits according to research.

### 3.1. Morphophysiological Characteristics of Crops

First of all, the morphophysiological parameters of tea plant callus cultures grown in the dark and in the light were studied, which makes it possible to assess their physiological state. In our case, cultures of 24 and 36 days of age (the beginning and end of the linear growth phase) were used, which, as was established earlier, are characterized by the most stable level of formation of specialized metabolites of a phenolic nature [56]. It was this aspect of their biosynthesis that was important for establishing their response to the impact of H_2_O_2_.

According to the data obtained, no changes in the morphology of callus cultures of tea grown under different conditions (darkness or light) were observed during exposure to H_2_O_2_ (Figure 1 and Figure 2). However, slight changes were observed during the DRCC, which were expressed by a darker color, and this effect was concentration-dependent. After exposure to H_2_O_2_, the water content of the cultures slightly decreased (by 5–10% relative to the control), which was more pronounced in 36-day-old cultures with DRCC. This indicates a slowdown in the processes of elongation and vacuolization of the cells of the tea callus cultures, due to the influx of H_2_O_2_ into them through the plasma membrane [14].

Thus, the exogenous effect of various H_2_O_2_ concentrations on the tea callus cultures grown in the dark and in the light did not affect their morphophysiological characteristics. This is probably due to the short-term entry of this molecule into cells and their successful “protection”, possibly due to PCs, which are substances with antioxidant activity [18,36].

### 3.2. Content of Malondialdehyde

It is known that the H_2_O_2_ molecule is an oxidative agent and, under stress conditions, it exhibits dual functions, acting as a toxic product on the one hand and a signal molecule on the other [67]. H_2_O_2_ entry into plant cells can lead to changes in their antioxidant status and the development of a stress response, the indicator of which is the level of lipid peroxidation, which is determined by the content of MDA. The level of lipid peroxidation (LPO) was higher in the tea cultures grown in the light, compared to that of the cultures grown in the dark, which was more pronounced on the 36th day of their growth (Figure 3). Perhaps this was due to the different levels of their differentiation and ultrastructure organization, which are important for LPO processes [8].

In 24-day-old tea cultures grown in the dark, the MDA content increased with 0.01 mM H_2_O_2_ and RRCC, and with 1 mM H_2_O_2_ and DRCC (by 25% relative to the control). Conversely, in 36-day-old cultures, the content of MDA increased only with the RRCC when the calli were exposed to 0.1 mM H_2_O_2_. One could also note an almost equal increase in the content of MDA in 24-day-old tea calli grown in the light with RRCC, with the effect of all the studied concentrations of H_2_O_2_ (by 15–17% relative to the control). This suggests that the cultures had the same response (stress) reaction to the intake of this molecule, regardless of the concentration used. In the 36-day-old cultures, an increase in the LPO level was noted only for the DRCC with 0.01 mM H_2_O_2_ (by 30% relative to the control). This indicated a pronounced post-effect of its impact and the absence of changes in the antioxidant system, which was typical for all other cultures.

All of the above confirms the results of other authors on the influence of plant cell cultivation conditions under in vitro conditions, their age, the duration of exposure (rapid and long-term response), and the H_2_O_2_ concentration on the processes of ROS formation and, as a result, on the LPO level [15,16,32].

### 3.3. The Total Phenolic Compounds

To control the level of ROS and prevent the formation of sequential LPO cascade reactions, an important role is played by low-molecular-weight components of the antioxidant defense system, which, along with tocopherols, carotenoids, proline, ascorbate, and glutathione, also include PCs [21]. Tea plants are among the crops with a high content of various PCs [42]. Their responses to the action of ROS may differ from those of other plants, where in most cases the stimulating effect of hormone-like concentrations of H_2_O_2_ was manifested [5,68].

In tea cultures grown in the dark, the content of total PCs did not change under the action of H_2_O_2_ on 24-day-old calli and almost doubled relative to the control in 36-day-old calli with the RRCC and DRCC for 0.1 mM H_2_O_2_ (Figure 4). In cultures grown in the light, the trends were different: in all experimental variants of 24-day-old calli, the total PC content was lower than in the control and almost equal for both the RRCC and DRCC (Figure 4b). To a certain extent, a similar situation was also characteristic of the 36-day culture, although with the DRCC it exceeded the control values for 1 mM H_2_O_2_. All this testifies to significant differences in the reaction of tea callus cultures grown in the dark and in the light to the exogenous effect of various H_2_O_2_ concentrations. This may be due to their stress response (dark-culture RRCC with 0.1 mM H_2_O_2_) or the redox signaling role of H_2_O_2_, when its appearance in the cell cytoplasm activates the MAPK (mitogen-activated protein kinase) cascade and leads to the activation of genes encoding for the enzymes of phenolic metabolism. There is a lot of data on the role of H_2_O_2_ in the formation of stress defense systems and on its redox regulatory component with a wide range of signaling and regulatory functions in the plant cell [5,67].

The above data indicate differences in the responses of tea callus cultures grown in the dark and in the light to the action of various H_2_O_2_ concentrations, and in the level of PC accumulation. This may be due to their endogenous content and participation in the protection of cells from ROS, as well as the level of intracellular differentiation, namely the absence/presence of chloroplasts, which are important sites for the biosynthesis of these specialized metabolites [10,19,69].

### 3.4. Flavan Content

Among the most common phenolic compounds in plants are flavonoids, which are able to effectively neutralize H_2_O_2_ diffusing into the vacuole during changes in the pro/antioxidant balance [70,71]. Their main components in tea plants and cultures initiated from them are FLs, which are substances with P-vitamin capillary-strengthening activity [46,62,72]. The accumulation of these PCs in callus cultures grown in the light significantly exceeded that of the cultures grown in the dark, which, apparently, contributed to differences in their response to H_2_O_2_ exposure (Figure 5). At the same time, in all cases, a clear relationship for the content of MDA as an indicator of the stress response was not observed, which may indicate the realization of the stress-protective properties of FLs. It is known that H_2_O_2_ at low concentrations can act as a component of redox signaling that was presumably manifested with 0.1 mM H_2_O_2_. An increase in the intracellular content of this molecule disrupts the processes of redox regulation and, as a result, the development of oxidative damage to macromolecules and cellular structures is also disrupted [5,67]. However, the levels of LPO in these variants are quite variable and, in some cases, do not correlate with changes in the content of PCs and FLs; therefore, the stress effect of a high concentration of H_2_O_2_ can only be assumed.

In the tea cultures grown in the light, the content of FLs on the 24th and 36th days of growth was more than 2 and 1.5 times higher than that of the cultures grown in the dark, respectively. This is due to the compartmentalization of flavonoid biosynthesis, as well as their photoprotective function under light conditions of plant growth [61]. Thus, the increased content of flavonoids in the epidermal cells allowed the cultures to absorb up to 95% of UV light, reducing the risk of photooxidative damage to cells by reducing the amount of ROS [7,73].

When H_2_O_2_ affected tea cultures of different ages grown in the light, the FL content either remained at the control level or decreased. The FL content was high in 36-day-old calli (by 40% relative to the control) only with the DRCC for 1 mM H_2_O_2_. It is possible that the initially high endogenous amount of FLs in the tea culture grown in the light was sufficient to realize their protective properties with regard to this molecule; therefore, no pronounced response was observed, which is consistent with the data on the content of MDA in callus cultures. The absence of a clear relationship between these parameters (FLs and MDA content) in tea callus cultures was probably determined by the experimental conditions, adaptive cell responses, exogenous action of H_2_O_2_, and the role of H_2_O_2_ in overall cell signaling and metabolism [7,9].

### 3.5. Content of Proanthocyanidins

The biosynthesis of PAs, which are flavan derivatives of various degrees of polymerization, is carried out at the final stages of the flavonoid pathway of phenol metabolism [63,65]. They are present in plant cells in the form of soluble (SPA) and insoluble (IPA) forms [65].

In tea callus cultures grown in the dark, changes in the content of SPAs under the action of H_2_O_2_ occurred only in 36-day-old cultures (Figure 6). It increased with the RRCC for 0.1 mM H_2_O_2_ (by 64% relative to the control) and with the RRCC for 0.1 mM H_2_O_2_ and 1 mM H_2_O_2_ (by 104% and 82%, respectively). Hence, the effect of H_2_O_2_ led to an increase in the formation of polymeric forms of flavans in tea cultures grown in the dark, but only at the end of the linear phase of their growth, that is, as the cultures aged. In tea calli grown in the light, the SPA accumulation decreased in almost all variants under exogenous exposure to H_2_O_2_. Their slight excess relative to the control was observed with the DRCC for 1 mM H_2_O_2_ in a 36-day-old callus.

As for the content of IPAs in callus tea cultures, their level was higher compared to SPAs, and this was more pronounced when the cultures were grown in the light (Figure 7). At the same time, the nature of their response to the effect of H_2_O_2_ in relation to the accumulation of these metabolites was similar to that for SPAs. All this allowed us to conclude that the formation of PAs (both SPAs and IPAs) in tea callus cultures depends on their age, the H_2_O_2_ concentration, and the duration of exposure. Changes in their accumulation, as well as in other PCs, reflected changes in the adaptive programs of plant cells, when specialized metabolites act as a target for H_2_O_2_ exposure and thus contribute to an increase in their resistance.

### 3.6. Lignin Content

Lignin or lignins are formed as a result of the dehydrogenation and polymerization of cinnamic alcohols, including with the participation of H_2_O_2_. They serve as candidates for the oxidation of monolignol structures and complete the assembly of these phenolic heteropolymers [43,74]. The processes of “crosslinking” of phenolic monomers during the oxidative binding of lignin subunits are necessary for the formation of cell walls, thereby slowing down the processes of cell growth and expansion. Lignin is also important for the formation of tracheal elements characteristic of tea callus cultures [46].

Considering the effect of H_2_O_2_ on the content of lignin in tea cultures grown in the dark and in the light, one can note the insignificant changes in lignin content, which occurred mainly during DRCC (Figure 8). In 24-day-old calli, in both cases, it was high with 1 mM H_2_O_2_; in 36-day-old calli, it was high with 0.01 mM H_2_O_2_. In the tea cultures grown in the light, the lignin content also increased with the RRCC for 1 mM H_2_O_2_, exceeding the control value by 30%. Based on these data, it can be assumed that a high concentration of this molecule has a regulatory effect on the formation of lignin in tea callus cultures. This indicates its important role in PC polymerization and lignin formation [43].

## 4. Materials and Methods

### 4.1. Tea Tissue Culture

Tea callus cultures established from the stems of young shoots of *Camellia sinensis* L. (strain IFR-ChS-2) were cultivated in the dark (no light exposure, intensity 0 lux) or in the light (16 h photoperiod, intensity 5000 lux) in a factorostat chamber at 26 °C and with a relative air humidity of 70% on Heller’s basic nutrient medium containing 2,4-dichlorophenoxyacetic acid (5 mg L^−1^), glucose (25 g L^−1^), and agar (7 g L^−1^) [56]. The passage duration was 46 days. When studying the action of H_2_O_2_, tea callus tissue at the age of 24 and 36 days (middle and end of the linear growth phase, respectively) was placed in sterile Petri dishes containing 20 mL of water (control) or an aqueous solution of H_2_O_2_ (concentrations of 0.01 mM, 0.1 mM, or 1 mM), and kept for 2 h. Further, part of the plant material was separated and used to study the rapid response of callus cultures (RRCC) to H_2_O_2_ exposure, while the other part was transferred to the agar nutrient medium and cultivated for 48 h, evaluating the post-effect of its exposure to H_2_O_2_ on callus cultures or their “delayed” response (DRCC). The H_2_O_2_ concentrations used in the experiments were adjusted earlier in preliminary studies. The material for biochemical analysis was fixed in liquid nitrogen and stored at −70 °C.

### 4.2. Determination of Morphophysiological Characteristics of Callus Cultures

The morphophysiological state of cultures was assessed by their external characteristics: color, callus density, and presence of necrotic areas. The fresh weight of calli was also determined.

### 4.3. Determination of Water Content in Callus Cultures

To determine the water content, 150 mg plant material samples were placed in glass weighing bottles and dried to a constant weight at 70 °C in the thermostat BD-115 (Binder, Tuttlingen, Germany) [75].

### 4.4. Determination of the Level of Lipid Peroxidation

The level of lipid peroxidation (LPO) in callus tea cultures was determined by the content of malondialdehyde (MDA) obtained in the reaction with thiobarbituric acid (TBA) [76]. A sample of the plant material frozen in liquid nitrogen was homogenized in 0.1 M Tris-HCl buffer (pH of 7.5) containing 0.35 M NaCl, after which 1 mL of a 0.5% TBA solution in a 20% aqueous solution of trichloroacetic acid was added. The reaction mixture was kept for 30 min in a water bath (100 °C) and then rapidly cooled. For the research, the supernatant obtained after the separation of the precipitate (16,000× *g*, 10 min) was used, with its optical density determined at 532 nm. To calculate the MDA content (µmol g^−1^ dry weight), the molar extinction coefficient (1.56 × 10^−5^ cm^−1^ M^−1^) was used [77].

### 4.5. Extraction of Phenolic Compounds from Plant Material

To extract PCs, plant material (50 mg) frozen in liquid nitrogen was homogenized in 96% ethanol and kept in a thermostat at 45 °C for 30 min in the dark [78]. The homogenate was centrifuged (16,000× *g*, 3 min). The supernatant fraction was used to determine various phenolic compounds, and the precipitate was used to determine the bound forms of proanthocyanidins.

### 4.6. Determination of the Total Content of Phenolic Compounds

The total PC content was determined using the Folin–Chiocalteu reagent [79,80]. An amount of 75 µL of the FC reagent was added to 75 µL of the ethanol extract or ethanol (control), and 150 µL of a 20% Na_2_CO_3_ solution and 1200 µL of distilled water were added after 3 min. The mixture was incubated at 24 °C for 30 min. The density of the solution was analyzed using spectrophotometry at 725 nm. Gallic acid was used to obtain the calibration curve. The total PC content was expressed in mg-equivalents of gallic acid g^−1^ dry weight.

### 4.7. Determination of Flavan Content

The content of flavans was determined with a vanillin reagent [81]. For this, 1250 µL of a 1% vanillin solution in 70% H_2_SO_4_ was added to 250 µL of the ethanolic plant extract [56]. As a control, a solution was used, where the plant extract was replaced by 96% ethanol. The optical density of the solution was analyzed using spectrophotometry at 500 nm. A calibration curve was obtained according to (−)-epicatechin. The content of flavans was expressed in mg-equivalents of epicatechin g^−1^ dry weight.

### 4.8. Determination of the Content of Soluble Proanthocyanidins

Soluble proanthocyanidins were determined using a butanol reagent [56,64]. A total of 1000 µL of the butanol reagent was added to 500 µL of the ethanolic plant extract, and the mixture was incubated at 95 °C for 45 min in the dark. In the control variant, the plant extract was replaced with 96% ethanol. The optical solution density was analyzed using spectrophotometry at 550 nm. The content of proanthocyanidins was expressed in mg-equivalents of cyanidin g^−1^ dry weight.

### 4.9. Determination of the Content of Insoluble (Bound) Proanthocyanidins

To determine the content of insoluble (bound) proanthocyanidins, the precipitate obtained after PC extraction from plant material was additionally washed with 96% ethanol, checking the absence of a reaction to flavans with vanillin reagent in the eluates [64]. An amount of 500 µL of 96% ethanol and 1000 µL of butanol reagent were added to precipitate, and incubated at 95 °C for 45 min in the dark. The optical density of the supernatant solution was analyzed using spectrophotometry at 550 nm. The content of proanthocyanidins was expressed in mg-equivalents of cyanidins g^−1^ dry weight.

### 4.10. Determination of Lignin Content

To determine the lignin content, the sample of plant tissue frozen in liquid nitrogen (50–100 mg) was successively extracted with 96% ethanol and a mixture of ethanol–benzene (1:2) in a Soxhlet apparatus for 6 h [24]. After that, the material was washed with water at 95 °C for 2 h. The precipitate was separated and washed three times with ethanol and ether. The extract-free material thus obtained was treated for 36 h with 0.5 N NaOH (2–3 mL) at 80 °C. The hydrolysate was cooled, centrifuged (16,000× *g*, 15 min), and diluted 10 times with water. An amount of 2 mL of 0.05 M Tris-HCl buffer (pH 9.0) and 0.25 mL of an ethanol solution of 2,6-dichloroquinone chlorimide (600 mg L^−1^) were added to 2.75 mL of the obtained solution [46]. The optical density of the solution was measured at 610 nm. The calibration curve was obtained according to ferulic acid.

### 4.11. Statistical Analysis

All determinations were carried out in three biological and three analytical replicates. The obtained data were statistically processed using Microsoft Excel 2010 14.0 (Redmond, WA, USA) and SigmaPlot 12.2 (Technology Networks, Sudbury, UK) software. The figures show the arithmetic means of the determinations and their standard errors (±SEM). The significance of differences in the mean values was determined by Tukey’s range test at *p* ≤ 0.05 and denoted by different Latin letters.

## 5. Conclusions

Plant cells and tissues cultivated in vitro are both producers of various metabolites and model systems for studying the influence of various exogenous factors, including those that increase their productivity. These include hydrogen peroxide (H_2_O_2_), which acts as a regulator, terminator, or stimulator of these processes, including the accumulation of biologically active substances. Among these unique substances are phenolic compounds (PCs), low-molecular-weight metabolites synthesized in almost all cells and characterized by high antioxidant activity. Of great interest in this aspect are tea plants (*Camellia sinensis* L.), which are widely used by the people of our planet in their daily diet and are important for health preservation. A characteristic feature of these cultures is the accumulation of various PCs, including flavans—compounds with P-vitamin capillary-strengthening activity. Other properties of tea phenolic metabolites are also known, including protective effects against COVID-19. The limited growing range of tea plants and their unique properties make it necessary to cultivate them under in vitro conditions, where, as was shown earlier, the ability to synthesize the PCs characteristic of the natural source was preserved.

In general, our results on the effect of H_2_O_2_ on tea plant callus cultures of different ages, grown in the dark or in the light, indicate their diverse response, which depends on the age of the culture and the growing conditions, as well as the time of studying the response (rapid or delayed effect). It should also be noted that signaling mechanisms do not work as separate linear pathways, since a complex of cross-reactions arises. The complex signaling network initiated by ROS in plants is controlled by various signaling mechanisms, and an analysis of the interaction of signaling pathways is significant for understanding fundamental physiological processes. It is important that signaling, which regulates the expression of genes for the biosynthesis of phenolic compounds, crosses with other signaling pathways, and therefore contributes to the integral response of the plant organism to various external influences.

The “double agent” role is typical for H_2_O_2_, which acts as stressor and regulator of biosynthetic processes. For this reason, we plan to conduct research on the regulatory role of H_2_O_2_ on other cultures that accumulate polyphenols to identify the features of this compound’s action on secondary metabolism processes.

## Data Availability

The data presented in this study are available on request from the corresponding author.

## Sample Availability

Samples of the compounds are not available from the authors.
